# Prolonged P Wave Peak Time May Be a Sign of LV Diastolic Dysfunction in the Coronary Slow Flow Phenomenon

**DOI:** 10.1155/2022/4626701

**Published:** 2022-01-31

**Authors:** Burhan Aslan, Ferhat Işık, Abdurrahman Akyüz, Ümit İnci, Muhammed Karadeniz

**Affiliations:** ^1^Diyarbakır Gazi Yaşargil Education and Research Hospital, Health and Science University, Department of Cardiology, Diyarbakır, Turkey; ^2^Kırıkkale University, Department of Cardiology, Kırıkkale, Turkey

## Abstract

**Background:**

The coronary slow flow phenomenon (CSFP) is an atherosclerotic process that causes ischemia at the microvascular level. The CSFP may affect P wave durations, especially P wave peak time (PWPT), by microvascular ischemia, left ventricular diastolic dysfunction, and changes in the left atrial dimension. Therefore, in the present study, we aimed to assess PWPT in the CSFP.

**Method:**

One hundred and ninety-five patients were included in this single-center, retrospective study. Ninety patients were enrolled in the CSFP group and 105 patients in the control group. PWPT was defined as the duration between the beginning and peak of the *p* wave and obtained from the leads *D*ıı and *V*_ı_.

**Results:**

The mean age of the study population was 48.5 ± 9.5, and 108 (55.3%) of the patients were female. We found PWPT was longer in the CSFP group than in the control group. Correlation analysis showed a positive correlation between PWPT in both leads (*D*_II_, *V*_ı_) and left atrial anterior-posterior diameter, mean TIMI frame count (TFC), and *E*/*e*. A significant relationship was observed between mean TFC, *E*/*e*, EF, heart rate, and PWPT in lead *D*_ıı_ (*β* coefficient = 0.33, 95% CI 0.44–1.33, *p* < 0.001, *β* coefficient = 0.23, 95% CI 0.25–1.85, *p*=0.01, *β* coefficient = -0.140, 95% CI −1.04–−0.53, *p*=0.03, and *β* coefficient = −0.13, 95% CI −0.29–−0.014, *p*=0.03, respectively) in multivariable linear analysis.

**Conclusion:**

In the present study, we found prolonged PWPT in patients with the CSFP and found a relationship between PWPT and mean TFC.

## 1. Introduction

The coronary slow flow phenomenon (CSFP) is defined as the slow progression of contrast in the absence of significant coronary artery stenosis [1]. Clinical presentation may be stable angina or acute coronary syndrome as a result of myocardial ischemia [2]. Many mechanisms may be responsible for this phenomenon, such as atherosclerosis, endothelial dysfunction, inflammation, small vessel involvement, and platelet dysfunction [3–5]. Previous studies have shown that the CSFP is an atherosclerotic process that causes ischemia at the microvascular level [4, 5]. Left ventricular (LV) diastolic dysfunction is an early clue for atherosclerotic coronary artery disease, and LVDD affects the left atrial (LA) volume and function [6]. LV diastolic dysfunction (LVDD) and changes in LA volume in patients with the CSFP have been shown in previous studies [7–10].

Electrocardiography (ECG) is the most important tool in the diagnosis of heart diseases. P wave peak time (PWPT) is a new parameter that can be detected from the ECG. Many P wave parameters have been obtained from the ECG, such as P wave dispersion (PWdıs), maximum P wave duration (PWDmax), and P wave terminal force from lead *V*_*I*_ (PWTF). They have been used for the assessment of LVDD and LA dilatation in studies [11, 12]. The correlation of left ventricular end-diastolic pressure (LVEDP) with PWPT has been shown in previous studies [13, 14]. Prolonged intra- and interatrial conduction time and elevated atrial pressure may manifest themselves as prolonged PWPT on ECG.

We considered the CSFP may affect P wave durations, especially PWTF and PWPT, by doing microvascular ischemia, LVDD, and changes in LA dimension. Therefore, this study aims to assess PWPT in patients with the CSFP.

## 2. Material and Method

### 2.1. Study Population

**Figure 1:**
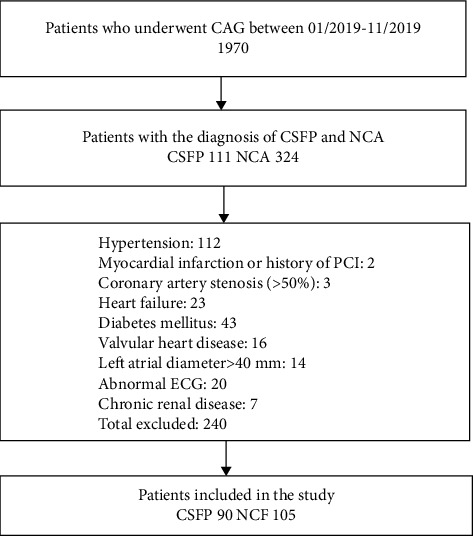
Flow chart of the study.

One thousand nine hundred and seventy patients were scanned from our hospital angiography laboratory between January 2019 and November 2019 for this single-center, retrospective study. One hundred and eleven patients were identified as the CSFP. The exclusion criteria were any of the following: hypertension, diabetes mellitus, history of percutaneous coronary intervention or myocardial infarction (MI), heart failure, coronary artery stenosis (>50%), moderate or severe valvular heart disease, LA anterior-posterior diameter >40 mm, atrial fibrillation, left or right bundle branch block, and acute/chronic kidney disease. After exclusion, 90 patients with the slow flow in at least one coronary vessel were included according to the criteria determined by Gibson et al. [15]. One hundred and five patients with normal coronary angiography results and without exclusion criteria were included as normal coronary flow (NCF). The study population consisted of patients who applied to the outpatient clinic due to chest pain or angina equivalent-like symptoms and underwent angiography due to a positive noninvasive test result (exercise test or myocardial perfusion imaging). Demographic data, medical histories, and laboratory findings were obtained from the electronic database of the hospital and the Turkish Ministry of Health. The study was approved by the ethics committee of our institution. A flow chart is shown in Figure 1.

### 2.2. Assessment of Electrocardiography

**Figure 2:**
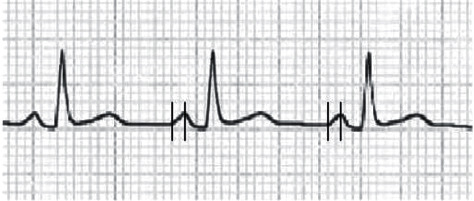
Measurement of *p* wave peak time in lead *D*_II_.

The ECG was recorded with a paper speed of 25 mm/s and an amplitude of 10 mm/mV for each patient. All ECG documents were scanned and investigated with a digital image program (imagej.nih.gov/ij). Analyses were performed by two observers who were blinded to the patients' data. the Bazett formula was used for calculating QTc intervals. P wave duration (PWDmax and PWDmin) was measured from all leads, and maximum and minimum values were recorded. P wave dispersion (PWdis) was obtained by subtracting maximum and minimum PWD. PWPT was defined as the duration between the beginning and peak of the p wave and obtained from the leads *D*ıı and *V*_ı_ [13] (Figure 2). P wave terminal force (PWTF) was obtained by multiplying the time and amplitude of the terminal negative component of the P wave in lead *V*_ı_, and ≥40 mm x ms was accepted as abnormal PWTF [12].

### 2.3. Assessment of Echocardiography and Coronary Angiography

Echocardiographic assessment of each patient was made before the procedure with the GE Vingmed Vivid S60N ultrasound system. Standard imaging techniques were performed in line with the American Society of Echocardiography guidelines [16]. Two-dimensional pulse Doppler, continuous-wave Doppler, color flow Doppler, and tissue Doppler measurement of mitral annulus were used. The modified Simpson method was used for measuring LV ejection fraction (EF). Pulse wave Doppler velocity was obtained from the apical window by placing the sampling volume on the mitral valve tips, and the images of three consecutive cycles were recorded. Mitral early peak velocity (E cm/s) and mitral late peak velocity (A cm/s) were evaluated. Tissue Doppler examination was performed on the apical four-chamber window by placing the sampling volume on the junction of the lateral annulus and the wall. Early diastolic velocity (e cm/s) and late diastolic velocity (A' cm/s) were calculated from the mitral lateral annulus with tissue Doppler evaluation. *E*/*A* and *E*/*e* ratios were calculated. The average of three consecutive measurements of each parameter was recorded. LA diameter was obtained by using parasternal long axis and apical four-chamber images. Echocardiographic evaluation was performed by an experienced observer.

Coronary angiography was performed via the femoral or transradial approach using standard techniques [17]. Coronary angiography was performed in patients with stable or unstable angina with myocardial ischemia detected by noninvasive tests. Thrombolysis in the myocardial infarction frame count (TFC) method was used for the assessment of coronary blood flow [15]. Measurements were performed by two observers who were blinded to the patients' data. Intraobserver agreement flow velocity was measured by observing the angiographic records taped at 15 square (15 fps) speed. The time when the contrast touches both sides of the artery and begins to forward motion down the artery was defined as the initial frame count. As the final frame count, the mustache was taken for the left anterior descending artery (LAD), the first distal longest branch was taken for the circumflex artery (CX), and the point where the posterior descending artery branch gave its first subbranch was taken for the right coronary artery (RCA). TFC was normalized by comparing the length of LAD with RCA and CX. Corrected TFC was obtained by dividing the total sine frame count of LAD by 1.7. The mean TFC was measured by adding the frame count for RCA, CX, and LAD and dividing the obtained value by three. Patients with at least one coronary artery with a frame number on the standard deviations of 20.4 ± 3.0 for RCA, 22.2 ± 4.1 for CX, and 36.2 ± 2.6 for LAD were determined as the CSFP [17].

### 2.4. Statistical Analysis

SPSS 22 package program was used for analysis. The distribution of variables was analyzed by using the Kolmogorov–Smirnov test. The Mann–Whitney *U* test or Student's *t*-test was used to compare continuous variables. The chi‐squared test or Fisher's exact test was used for categorical variables. The Pearson test was used for the correlation analysis. Data are expressed as percentages for categorical variables and as mean ± SD for parametric variables. Multivariable linear regression analysis was utilized to assess the relationship between age, gender, LA diameter, *E*/*e*, EF, heart rate, mean TFC, and PWPT. All parameters were evaluated with the PWPT in lead *D*_ıı_ and *V*_ı_ separately. The interobserver and intraobserver variability for ECG measurements were assessed by intraclass correlation coefficients (ICCs) using a model of absolute agreement. ICC greater than 90 indicates excellent agreement, 75–90 good, 50–74 moderate, and <50 poor agreement [18]. Values of *p* < 0.05 were considered statistically significant.

## 3. Results

One hundred and ninety-five patients were included in the present study. The study consisted of two groups as the CSFP (*n* = 90) and NCF (*n* = 105). The mean age of the patients was 48.5 ± 9.5, and 108 (55.3%) of the patients were female. There was no statistical difference between the two groups in terms of the medications. Left atrial anterior-posterior diameter (LAAPD) (36.6 ± 3.5 vs. 33.9 ± 2.6, *p* < 0.001) and *E*/*e* (9.4 ± 2.1 vs. 6.4 ± 1.4, *p* < 0.001) were higher in the CSFP group than in the NCF group. E/A (0.85 ± 0.23 vs. 1.1 ± 0.38, *p*=0.001) and *E* wave velocity (0.67 ± 0.21 vs. 0.82 ± 0.24, *p*=0.001) were statistically lower in the CSFP group compared to the NCF group. The demographic features and laboratory findings of the groups are listed in Table 1.

There was good intraobserver and interobserver agreement for PWPT in lead D_II_ and *V*_1_ (ICC = 0.90, 95% CI 0.80–0.95, ICC = 0.88, 95% CI 079–0.94, ICC = 0.86, 95% CI 0.75–0.92, and ICC = 0.80, 95% CI 0.63–0.86, respectively). Also, there was good intraobserver and interobserver agreement for PWTF and PWdis (ICC = 0.84, 95% CI 0.76–0.92, ICC = 0.74, 95% CI 0.68–0.87, ICC = 0.83, 95 CI 0.74–0.89, and ICC = 0.76, 95% CI 0.67–0.84, respectively).

PWDmax (121 ± 11 vs. 103 ± 12, *p* < 0.001), PWDmin (80 ± 14 vs. 74 ± 11, *p*=0.002), and PWdisp (40 ± 11 vs. 29 ± 9, *p* < 0.001) were longer in the CSFP group compared to the NCF group.

**Figure 3:**
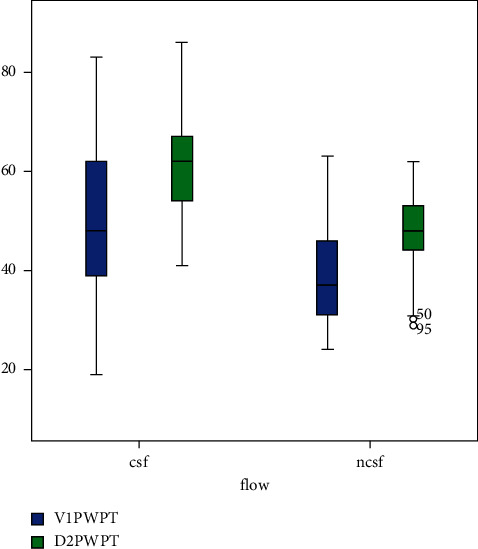
Comparison of the PWPT in lead *D*2 and *V*1 between CSFP and NCF groups. PWPT in lead *V*_I_ and PWPT in lead *D*_II_ were significantly longer in the CSFP group compared to the NCF group (*p* < 0.001, *p* < 0.001, respectively).

PWTF was abnormal (≥40mmxms) in CSFP patients. PWPT in lead *V*_I_ (51 ± 14 vs. 39 ± 9, *p* < 0.001) and PWPT in lead D_II_ (61 ± 9 vs. 48 ± 7, *p* < 0.001) were significantly longer in the CSFP group. As expected, TFC for all coronary arteries was higher in the CSFP group. ECG and angiographic findings of groups are listed in Table 2 and Figure 3.

Subgroup analysis of the CSFP group is given in Table 3. The CSFP group was divided into two groups according to multivessel and single vessel in terms of slow flow. PWPT in lead *V*_I_ (48 ± 14 vs. 55 ± 14, *p*=0.029) was longer in the multivessel group than in the single-vessel group. LAAPD (35 ± 2 vs. 39 ± 3, *p* < 0.001) and *E*/*e* (8.8 ± 1.4 vs. 10.7 ± 2.7, *p*=0.001) were higher in the multivessel subgroup compared to the single-vessel subgroup.

Correlation analysis showed a positive correlation between PWPT in both leads (*D*_II_, *V*_I_) and LAAPD, mean TFC, and *E*/*e*. Also, a positive correlation was observed between PWTF and LAAPD, mean TFC, and *E*/*e*'. Correlation analysis is shown in Table 4.

A significant relationship was observed between mean TFC, *E*/*e*, EF, heart rate, and PWPT in lead *D*_ıı_ (*β* coefficient = 0.33, 95% CI 0.44–1.33, *p* < 0.001, *β* coefficient = 0.23, 95% CI 0.25–1.85, *p*=0.01, *β* coefficient = −0.140, 95% CI −1.04–−0.53, *p*=0.03, and *β* coefficient = −0.13, 95% CI −0.29–−0.014, *p*=0.03, respectively). Also, a significant relationship was observed between mean TFC, *E*/*e*, and PWPT in lead *V*_ı_ (*β* coefficient = 0.29, 95% CI 0.39–1.59, *p*=0.01; *β* coefficient = 0.21, 95% CI 0.11–2.33, *p*=0.03, respectively). Multivariable linear regression analysis is listed in Table 5.

## 4. Discussion

Our study demonstrated that the PWPT is longer in CSFP patients compared to the NCF patients. There was a significant relationship between mean TFC and PWPT in both leads *D*_ıı_ and *V*_ı_. And also, PWPT in lead *V*_ı_ was longer in the multivessel CSFP group compared to the single-vessel CSFP group.

CSFP incidence ranged between % 1–7 in performed coronary angiographies [19]. To date, the underlying mechanism is not known exactly. Patients with the CSFP may present with recurrent anginal chest pain, arrhythmia, and unexpected sudden cardiac events [20], and there is no consensus about the treatment. This situation encourages clinicians to conduct more research on the CSFP. There are different results according to the studies about the effects of the CSFP on diastolic function. Fallah et al. evaluated LA strain and strain-rate parameters in the CSFP group, and they found reservoir, conduit, and pump function of LA were similar between the groups [7]. In contrast, Wang et al. and Sezgin et al. found LV diastolic dysfunction in patients with the CSFP [8, 9]. In this study, we found that *E*/*e* and LAAPD were higher and *E*/*A* and *E* velocity were lower in CSFP patients compared to the normal group. An increase in parameters such as *E*/*e* and LAAPD and a decrease in *E*/*A* and *E* velocity may show high left ventricular filling pressures and impaired diastolic function.

LA has 3 functions: reservoir function, conduit function, and pump function [21]. The conduit function comprises 2 phases: the passive discharge phase (fast filling phase) and diastasis phase. The passive phase depends on the relaxation, compliance, and viscoelastic properties of the myocardium. The diastasis phase usually depends on LV compliance [21]. LV compliance may decrease due to ischemia in patients with the CSFP [8]. Xing et al. evaluated LA volume and function by using three-dimensional echocardiography. Three-dimensional echocardiography showed higher LAV-max and LAV-min in the CSFP group compared to the normal coronary flow group. They found positive correlations between mean TFC values and LAV-max and LAV-min [22]. In our study, we found higher LAAPD in CSFP patients compared to the control group, and there was a positive correlation between mean TFC and LAAPD.

ECG is still an effective diagnostic tool in cardiovascular science despite all the developments. Many P wave abnormalities in ECG may provide clues for many cardiac diseases. Left atrial remodeling may show itself as prolongation in PWD and increase in PWdisp on ECG, and this remodeling may develop in acute and chronic events. PWD and PWdisp have been evaluated in diseases such as coronary artery disease, hypertension, atrial fibrillation, and CSFP. Akdemir et al. found prolonged PWD and increased PWdisp in acute myocardial infarction [23]. Turkmen et al. showed prolongation of PWD and increased PWdisp in CSFP patients [24]. These studies attributed these results to ischemia or partial reperfusion. In patients with the CSFP, ischemia is caused by endothelial dysfunction, vasoconstriction, and atherosclerosis in the microvascular area. Due to ischemia, the CSFP may cause diastolic dysfunction and, thus, *p* wave abnormalities. In our study, we found similar findings, such as prolonged PWD and increased PWdisp in the CSFP group.

PWPT and PWTF are new *p* wave parameters on ECG. They have been studied in acute coronary syndrome, hypertension, and paroxysmal atrial fibrillation. PWTF is a marker showing left atrial abnormalities such as increased left atrial volumes and elevated left atrial filling pressure [25]. A recent study demonstrated that abnormal PWTF is associated with impaired diastolic function in hypertensive patients [12]. Another study showed a relationship between abnormal PWTF and increased LV end-diastolic pressure [14]. Yıldırım et al. found that abnormal PWTF is associated with atrial fibrillation [26]. In the present study, PWTF was significantly abnormal (≥40 mmxms) in CSFP patients and correlated with mean TFC, *E*/*e*, and LAAPD.

Cengiz et al. showed a correlation between PWPT and increased left ventricular end-diastolic pressure and left atrial volume in patients with hypertension. They demonstrated prolonged PWPT may predict LVDD [14]. Çağdaş et al. showed a correlation between prolonged PWPT and coronary no-reflow in patients with the acute coronary syndrome [13]. Coronary no-reflow is defined as failure to restore myocardial reperfusion after primary PCI at the microvascular level despite optimal coronary patency. It has been known that microvascular dysfunction, tissue edema, and vasoconstriction are the underlying mechanism of no-reflow [27]. Microvascular dysfunction, endothelial dysfunction, and vasoconstriction are also the underlying mechanisms of the CSFP. Cengiz and colleagues showed an association between prolonged PWPT and the severity of coronary artery disease in patients with acute coronary syndrome [28]. In these studies, they explained that LA electrical and mechanical changes due to coronary ischemia may cause these ECG abnormalities. Based on these findings, we thought that the CSFP might affect PWPT by causing ischemia-induced LVDD and changing the LA dimension. In our study, PWPT in leads *D*_ıı_ and *V*_ı_ was significantly longer in CSFP patients. We found a significant positive correlation between PWPT in both leads (*D*_ıı_ and *V*_ı_) and mean TFC, *E*/*e*, and LAAPD. Furthermore, Ciccone et al. found that age and sex have an impact on endothelial dysfunction and age has been linked to progressive endothelial dysfunction, which occurs earlier in men than in women [29]. In the present study, there was no statistical significance in terms of age and gender between groups. There was no relationship between age, gender, and PWPT. In subgroup analysis, PWPT in lead *V*ı was longer, and *E*/*e* and LAAPD were significantly higher in the multivessel slow flow group compared to the single-vessel group. These findings can be explained by the larger myocardial area and ischemia affected by the multivessel slow flow.

## 5. Study Limitations

There are a few limitations to our study. The major limitations are that it is a retrospective and single-center study. Some parameters may not have been fully documented due to the retrospective design of our study, and the single-center nature of our study may reduce the effect of different patient populations on results. The small patient population is another limitation of the study. Using LA volume and index instead of LAAPDD would have given more accurate results. Therefore, prospective studies with more patients are needed on this subject.

## 6. Conclusions

In the present study, we found prolonged PWPT in patients with the CSFP and the relationship between PWPT and mean TFC. Prolonged PWPT may be a finding of LVDD on ECG in CSFP patients and can be used to distinguish patients who need close follow-up and treatment.

## Figures and Tables

**Table 1:** The baseline characteristics and laboratory and echocardiographic findings of groups.

Variables	CSFP (*n* = 90)	NCF (*n* = 105)	*p* value
Age	49.3 ± 9.7	47.6 ± 9.3	0.16
Female sex; *n* (%)	49(54.4)	59 (56.1)	0.09
Smoking; *n*(%)	30 (33.3)	28 (26.6)	0.29
Beta-blocker; *n* (%)	35 (38.8)	42 (40)	0.37
Non-DHD CCB; *n* (%)	23 (25.5)	20 (19)	0.46
Heart rate;/min	72 ± 9	74 ± 8	0.08
Fasting glucose level; mg/dl	105 ± 22	105 ± 24	0.99
Creatinin; mg/dl	0.75 ± 0.1	0.71 ± 0.1	0.08
White blood cell count; 10/*µ*l	9.1 ± 3.5	8.5 ± 2.2	0.16
Platelet count; 10^3^/*µ*l	240 ± 51	296 ± 73	**<0.001**
Haemoglobin; gr/dl	14.9 ± 1.5	13.8 ± 1.8	**<0.001**
Total cholesterol; mg/dl	176 ± 36	181 ± 39	0.36
Triglyceride; mg/dl	190 (119–251)	182 (102–228)	0.26
HDL-C; mg/dl	40 ± 9	44 ± 15	**0.027**
LDL-C; mg/dl	101 ± 29	104 ± 30	0.59
LVEF; %	59.3 ± 2.8	59.2 ± 2.5	0.88
LAAPD; mm	36.6 ± 3.5	33.9 ± 2.6	**<0.001**
*E*/*e*	9.4 ± 2.1	6.4 ± 1.4	**<0.001**
*E*/*A*	0.85 ± 0.23	1.1 ± 0.38	**0.001**
*E*	0.67 ± 0.21	0.82 ± 0.24	**0.001**

Values are mean ± SD (only triglyceride values are expressed as ınterquartile range). Bold values are statistically significant (*p* < 0.05). CSFP, coronary slow flow phenomenon; NCF, normal coronary flow; LAAPD, left atrial anterior-posterior diameter; LVEF, left ventricular ejection fraction; HDL, high-density lipoprotein; LDL, low-density lipoprotein; non-DHD CCB, nondihydropyridine calcium channel blocker.

**Table 2:** Angiographic and electrocardiographic findings of groups.

Parameters	CSFP (*n* = 90)	NCF (*n* = 105)	*p* value
QT; ms	385 ± 36	377 ± 27	0.06
QTc; ms	409 ± 29	408 ± 22	0.76
PWDmax; ms	121 ± 11	103 ± 12	**<0.001**
PWDmın; ms	80 ± 14	74 ± 11	**0.002**
PWdısp; ms	40 ± 11	29 ± 9	**<0.001**
PW morphology in lead *V*_ı_			
Negative; *n* (%)	25 (25.8%)	24 (23.3%)	
Positive; *n* (%)	29 (29.9%)	35 (34%)	0.81
Biphasic; *n* (%)	43 (44.3%)	44 (42.7%)	
PWTF in lead *V*_I_; mm xms	38 ± 7	28 ± 8	**<0.001**
PWPT _VI_; ms	51 ± 14	39 ± 9	**<0.001**
PWPT _DII_; ms	61 ± 9	48 ± 7	**<0.001**
TIMI-frame LAD	37.1 ± 5.0	21.1 ± 1.7	<**0.001**
TIMI-frame CX	26.7 ± 2.9	18.1 ± 1.6	**<0.001**
TIMI-frame RCA	25.1 ± 2.9	16.4 ± 1.8	**<0.001**
Mean frame count	27.2 ± 3	19.7 ± 2.0	**<0.001**

Values are mean ± SD; bold values are statistically significant (*p* < 0.05). CSFP, coronary slow flow phenomenon; NCF, normal coronary flow PWD, *p* wave duration; PWdısp, *p* wave dispersion; PWTF, *p* wave terminal force; PWPT, *p* wave peak time; QTc; corrected QT; TIMI, thrombolysis in myocardial infarction.

**Table 3:** Subgroup analyses of the CSFP group.

Parameters (*n* = 32)	Single vessel (*n* = 58)	Multi vessel (*n* = 32)	*p* value
QT; ms	383 ± 37	389 ± 34	0.41
QTc; ms	407 ± 27	414 ± 33	0.37
PWDmax; ms	121 ± 11	120 ± 10	0.64
PWDmın; ms	80 ± 13	80 ± 14	0.90
PWdısp; ms	41 ± 10	39 ± 12	0.67
PWTF in lead *V*_I_; mm xms	39 ± 7	38 ± 6	0.60
PWPT_V1_; ms	48 ± 14	55 ± 14	**0.02**
PWPT _D2_; ms	61 ± 9	60 ± 10	0.95
LAAPD; mm	34 ± 2	37 ± 2.3	**<0.001**
*E*/*e*	8.8 ± 1.4	10.7 ± 2.7	**0.001**

Values are mean ± SD. Bold values are statistically significant (*p* < 0.05). QTc, corrected QT; PWD, *p* wave duration; PWdısp, *p* wave dispersion; PWTF, *p* wave terminal force; PWPT, *p* wave peak time; LAAPD, left atrium anterior-posterior diameter.

**Table 4:** Correlation analysis of *p* wave parameters.

	Mean TFC	LAAPD	*EE*/*e*
PWTF *V*_1_			
*r* value	0, 52	0.35	0.44
*p* value	**0.001**	**0.001**	**<0.001**

PWPT *D*_2_			
*r* value	0.46	0.38	0.5
*p* value	**<0.001**	**0.001**	**<0.001**

PWPT *V*_1_			
*r* value	0.36	0.32	0.44
*p* value	**<0.001**	**0.001**	**<0.001**

Bold values are statistically significant (*p* < 0.05). PWTF, *p* wave terminal force; PWPT, *p* wave peak time; LAAPD, left atrium anterior-posterior diameter; mean TFC, mean TIMI frame count.

**Table 5:** Multivariable linear regression analysis.

Variables		PWPT in lead D2			PWPT in lead V1	
	*β* coefficient	(95% CI)	*p* value	*β* coefficient	(95% CI)	*p* value
Mean TFC	0.33	0.44–1.33	**<0.001**	0.29	0.39–1.59	**0.01**
E/*e*	0.23	0.25–1.85	**0.01**	0.21	0.11–2.33	**0.03**
EF	−0.14	−1.04–−0.053	**0.03**	−0.04	−0.91–0.43	0.48
LAAPD	0.07	−0.74–0.24	0.33	−0.02	−0.78–0.58	0.77
Age	0.08	−0.48–0.24	0.18	0.13	−0.01–0.38	0.06
Gender, male	0.09	−0.60–4.70	0.13	−0.08	−5.81–1.35	0.22
Heart rate	−0.13	−0.29–−0.01	**0.03**	−0.05	−0.27–−0.10	0.36
Beta-blocker	−0.03	−4.23–2.60	0.64	−0.02	−5.55–3.80	0.79
Non-DHD CCB	−0.03	−4.71–2.78	0.62	−0.02	−5.03–3.60	0.70

Bold values are statistically significant (*p* < 0.05*P* < 0.05). TFC, TIMI frame count; PWPT, *p* wave peak time; EF, ejection fraction; LAAPD, left atrial anterior-posterior diameter; non-DHD CCB, nondihydropyridine calcium channel blocker.

## Data Availability

The data used to support the findings of this study are restricted by the Ethics Board of the Gazi Yasargil Education and Research Hospital in order to protect patient privacy. Data are available from the corresponding author for researchers who meet the criteria for access to confidential data upon request.
